# New Paradigms in the Pathogenesis of Otitis Media in Children

**DOI:** 10.3389/fped.2013.00052

**Published:** 2013-12-23

**Authors:** James Mark Coticchia, Michael Chen, Livjot Sachdeva, Sean Mutchnick

**Affiliations:** ^1^Department of Otolaryngology – Head and Neck Surgery, Wayne State University School of Medicine, Detroit, MI, USA; ^2^Wayne State University School of Medicine, Detroit, MI, USA

**Keywords:** otitis media, otitis media with effusion, acute otitis media, biofilms, pathogenesis, middle ear infection

## Abstract

Acute otitis media (AOM) is a multifactorial disease with a significant socioeconomic impact. The pathogenesis of AOM is attributed to a variety of well-established internal and extrinsic factors. Recent evidence strongly points to bacterial biofilm formation as an important contributor to this disease entity. The nasopharynx is a likely reservoir for infection with subsequent seeding of pathogens to the middle ear via planktonic shedding. Various modalities have been used to directly detect biofilm formation in the middle ear mucosa of children with AOM. Further insights into this disease may lead to new strategies for prevention and treatment.

## Introduction and Epidemiology

### Prevalence and socioeconomic impact

Otitis media (OM) is one of the most common childhood infections. Clinically it is characterized by middle ear effusion (MEE) and recognized as acute otitis media (AOM) or OM with effusion (OME) (1, 2). OM is the leading reason for visiting the doctor, prescribing antibiotics, and undergoing surgical procedures among children (1, 3–6).

The peak incidence of AOM is between 6 and 12 months of age (7, 8). More than 80% of children are diagnosed with AOM by age 3 (7). National medical expenditures for OM have been estimated at approximately $4.1 billion for children (9, 10). While the incidence of OM in the U.S. rose steadily in the latter part of the twentieth century (8), there has been decline in the number of pediatric office visits between 1997 and 2007 (11). Rates of recurrent AOM (RAOM, defined in the study as >3 episodes in the previous 12 months) also declined between 2001 and 2005 (11).

The declining incidence of OM may be attributed to a number of factors that have been the focus of public health and education. The 7-valent pneumococcal conjugate vaccine (PCV7) was introduced in 2000 and has excellent efficacy against invasive pneumococcal disease (12–14). The “watchful waiting” guidelines aim to prevent unnecessary physician consultation for mild OM (14). Reductions in known risk factors such as smoke exposure may further contribute to the decline (14, 15).

### Widespread antibiotic use and increasing bacterial resistance

Use of broad-spectrum antimicrobials in the United States has increased over the last several decades (15, 16) and is directly correlated to rising antibiotic resistance (17, 18). Decreasing susceptibility of invasive *S. pneumonia* – the most common cause of pediatric AOM – to penicillin, clindamycin, and macrolides began in the mid-1990s (15, 19–21).

The serotypes covered by PCV7 were selected to protect against strains prominent among children worldwide and strains likely to develop antimicrobial resistance (22). The decrease in prevalence of PCV7-related *S. pneumoniae* has been met with increasing prevalence of PCV7-unrelated *S. pneumoniae* (23, 24) and *H. influenzae* (4, 25).

Selective pressure from the inappropriate use of antimicrobial agents is the single greatest factor influencing the spread of resistant *S. pneumoniae* and other common middle ear pathogens (15, 18, 20, 23, 25). Despite efforts by the Center for Disease Control (CDC) to minimize inappropriate use of antimicrobials, these practices continue to select for resistant pathogens. Strict guidelines for the diagnosis and treatment of OM are imperative.

## Diagnosis, Signs, and Symptoms

Children may present with non-specific findings such as ear-tugging, irritability, fever, or symptoms of a viral illness (3). Symptom duration and severity are not reliable indicators of AOM (3, 26). Accurate diagnosis of AOM requires a thorough examination of the tympanic membrane (TM) (3).

Recent updates to the AAP/AAFP guidelines for the diagnosis and treatment of AOM in children have narrowed down the diagnostic criteria, particularly in its distinction from OME (3). Diagnosis requires the presence of MEE with acute onset of signs and symptoms of middle ear inflammation. MEE is established by an air-fluid level behind the TM, impaired TM mobility on pneumatic otoscopy, or otorrhea signifying TM perforation. AOM is reliably distinguished from OME by the addition of a “cloudy” and moderate-to-severe bulging of the TM; less specific signs include significant TM erythema or hemorrhage (3, 27, 28). Additional methods for confirming MEE include tympanometry, acoustic reflectometry, or tympanocentesis.

## Risk Factors

### Host

AOM is most common in infancy and early childhood, with peak incidence between 6 and 12 months of age (7, 8). The immature state of the immune system in young children predisposes them to infection, particularly with encapsulated bacteria (29). Incidence tends to be higher in males (7, 30, 31).

The etiology and pathogenesis of AOM are multifactorial and represent the interplay between genetic and environmental factors (11). Twin studies have shown that heritability accounts for 74 and 45% of variation in RAOM incidence in females and males, respectively (29). Several indigenous populations are high-risk for OM: Native Americans, the Alaskan, Canadian and Greenland Inuit, and Australian Aborigines (11, 32). Recent availability of genome-wide association studies (GWAS) has greatly expanded the ability to search for related genes (30).

The Eustachian tube (ET) helps maintain healthy middle ear conditions. The ET of infants in relation to their fully matured anatomy is of a smaller caliber, shorter length, and joins the nasopharynx at a more acute angle, all of which predispose to dysfunction of the ET and therefore increased risk of infection (31, 33). Children with craniofacial anomalies, such as cleft palate and Trisomy 21, are at increased risk of middle ear disease due to further ET compromise (34–37). Histopathology studies have reported deformed ET cartilage (38) and high incidence of OM (36) in patients with cleft palate.

Some studies have postulated that atopic diseases such allergic rhinitis and asthma can play a role in OM (39). This may be due to increased susceptibility to invasive pneumococcal disease. However, the exact causal relationship has yet to be elucidated.

### Environment

Upper respiratory tract infections (URTI), both viral and bacterial, have been implicated in the development of AOM due to mucosal inflammation leading to adenoid hypertrophy, ET dysfunction, and disruption of mucociliary defenses (31, 40, 41). Virus-mediated inflammatory responses in the middle ear impair host immunity, promote bacterial colonization and inhibit antimicrobial penetration into the middle ear (31, 40). A temporal relationship is consistently observed with AOM incidence peaking 3–4 days after the onset of URTI symptoms (40).

Exposure to tobacco smoke is well known to adversely affect the respiratory tract. Higher colonization by pathogenic bacteria has been demonstrated in the nasopharyngeal flora of smokers and smoke-exposed children (42). Despite this, studies have not found a consistent link between smoke exposure and AOM incidence (43, 44). Potential confounding by socioeconomic status, thought to be inversely correlated with household smoking, often complicates the interpretation of such studies (43).

The protective effect of breastfeeding on OM incidence has been reported by the majority of investigators (43, 45). Proposed explanations for these protective effects have included head positioning during feeding, exposure to different microorganisms, improved nutrition and the antibacterial or immunological benefits of breast milk (46).

Exposure to other children, whether at day care or to siblings at home, is a strong risk factor for OM (11, 31, 43, 44, 46–48). Specific predictors include attendance by 2 months of age, attendance ≥30 h/week, and day care groups with ≥5 children and ≥2 children 2 years or younger (46).

## Classical Theory of Pathogenesis

Classical theories of OM pathogenesis describe MEE and subsequent infection as direct consequences of ET dysfunction. The ET preserves normal middle ear conditions through three primary functions: clearance of middle ear fluid (MEF), ventilation, and protection from nasopharyngeal reflux (49, 50).

The drainage system of the middle ear is eloquently portrayed as an inverted flask by Bluestone et al. with the body of the flask representing the middle ear and the narrow neck representing the ET (51). The mucociliary wave transports middle ear secretions toward the nasopharynx. When a precipitating event, such as viral URTI or allergic rhinitis, triggers nasal mucosal inflammation, obstruction of the tubal orifice leads to fluid stasis. Individuals with narrower and horizontally oriented anatomy, cleft palate, or tensor veli palatini deficiency have impaired drainage and are at higher risk for MEE (51).

In the open state, the ET ventilates the middle ear and equalizes pressure with the nasopharynx. This can be done deliberately with the Valsalva maneuver or palatal elevation via the action of tensor veli palatini. Studies have reported middle ear gas absorption at a constant rate of 1 mL/24 h (52, 53), The hydrops ex vacuo theory, original proposed by Politzer, postulates that continuous negative middle ear pressure causes transudation of fluid from mucosa into the middle ear cavity leading to effusion (54). The hydrops ex vacuo theory has been validated in human studies (55, 56) and is widely accepted as a key step in the development of MEE.

The et allows greater physical separation between the nasopharynx and middle ear, isolating the middle ear from infection and offensive material originating from the upper aerodigestive tract. This is believed to play a role in the higher incidence of OM in children, who have smaller and more horizontal ET compared to adults. Higher rates of gastroesophageal reflux, confirmed by detection of pepsin/pepsinogen in MEF, have been reported in children with OME or RAOM compared to otherwise healthy children (57). Pepsinogen in the middle ear has also been identified in the adenoids of children with OME, suggesting nasopharyngeal reflux as the likely mechanism (58). While some prospective studies have reported a possible benefit in OM resolution with antireflux therapy, evidence from large controlled trials is lacking (57, 59). There are currently no recommendations for the use of antireflux therapy in treating OM.

## The Importance of Biofilm Phenotypes in Otitis Media

### The chinchilla model

The vast majority of animal models of OM have utilized the chinchilla. Giebink cites several factors favoring these animals in the study of middle ear disease: (1) it is the only animal model in which *S. pneumoniae* OM can be induced by inoculation directly into the middle ear or nasal cavity, (2) infection rarely spreads outside of the middle ear, (3) the middle ear is easily accessible for inoculation and culture, and (4) OM does not naturally occur in chinchillas (60). Research on chinchilla models, beginning in the 1970s, have contributed to the discovery of potent *S. pneumoniae* serotypes (61), identify a role for nasopharyngeal viral infection in OM pathogenesis, and confirm an immunogenic response to pneumococcal vaccination (62). Today, the chinchilla model remains the cornerstone of basic science research in OM.

### Nasopharyngeal bacterial colonization

Nasopharyngeal colonization with potential middle ear pathogens is regarded as the initial event leading to OM in humans (63, 64). This theory is strongly supported by work on chinchilla models (65), which have shown a close correlation between nasopharyngeal and middle ear pathogens known to cause AOM (66). At the same time, children without RAOM carry greater species of benign nasopharyngeal flora which are thought to inhibit colonization and proliferation of pathogenic species (67). Many surgeons now routinely obtain middle meatus or nasopharyngeal cultures in children with RAOM to identify the causative agents and guide antimicrobial therapy.

### Biofilms and planktonic shedding

Biofilms are increasingly recognized as a key component of many chronic and treatment-resistant diseases. Hall-Stoodley et al. define biofilms as “surface-associated microbial communities surrounded by an extracellular polymeric substance matrix” which are notoriously resistant to host immune responses and antimicrobial therapy (68, 69). Properties of biofilms favoring their survival include (1) poor antimicrobial penetration, (2) decreased oxygen and nutrient requirements, (3) increased expression of resistance genes (e.g., beta-lactamase), and (4) cell-to-cell signaling via quorum sensing (70, 71). The extracellular matrix confers reduced permeability to topical and intravenous antimicrobials and along with other putative resistance mechanisms of biofilms (slower growth rate, oxygen depleted microenvironment, and other environmental stresses due to altered physiologic conditions) accounts for the frequent failure of traditional therapies (70). Quorum sensing involves intercellular transmission of molecules and genetic information which permit coordinated behavior and reaction to the local environment (71). Extensive research efforts to understand the biofilm environment have identified a number of therapeutic targets and will be discussed in the next section.

An overwhelming majority of bacteria in the human body exist in the biofilm state during which they are extremely difficult to culture (72). Bacterial biofilms have been implicated in chronic rhinosinusitis (*Staphylococcus aureus*) (73), recurrent UTI (*E. coli*), and cystic fibrosis pneumonia (*Pseudomonas aeruginosa*) (68, 71). It is likely that patients with impaired airway clearance are at higher risk for biofilm formation (71), although differences in structure or behavior are unclear. The observation that RAOM often results in negative MEF cultures and recurs despite appropriate antimicrobial therapy has driven the search for biofilms in the middle ear and nasopharynx (74).

Prior studies using chinchilla models led to direct confirmation of middle ear mucosal biofilms in the setting of OM through scanning electron microscopy (SEM) (75, 76), and confocal scanning laser microscopy (CSLM) (76). Biofilms were later confirmed in the middle ear mucosa of children with chronic OM (77, 78), and on the surface of tympanostomy tubes extracted from children with RAOM (75) and OME (79). None of these studies identified significant biofilms in healthy controls, strongly suggesting a role for biofilms in RAOM pathogenesis. Hoa et al. reported that within 8 days of nasopharyngeal inoculation with influenza A virus and *S. pneumonia* in chinchillas, 83% of animals developed nasopharyngeal biofilms, 67% developed middle ear biofilms, and all animals with middle ear biofilms also had nasopharyngeal biofilms (80). Hoa suggested that the presence of nasopharyngeal biofilms may be a prerequisite for development of biofilms in the middle ear.

Within the nasopharynx, the adenoids are a likely reservoir for pathogenic bacterial biofilms (64, 81–83). Adenoid biofilms may be particularly critical in the pathogenesis of RAOM compared to OME. Zuliani et al. and Hoa et al. demonstrated significantly greater biofilm coverage of adenoid mucosa in children with RAOM compared to children with OME or OSA (81, 82).

A unique feature of biofilms is “planktonic shedding” of bacteria from the biofilm surface into the surrounding space, seeding infection to distant parts of the body in a fashion similar to septic emboli (81, 84, 85). Planktonic shedding is a continuous process which appears to escalate during conditions of physiological stress and starvation, leading Costerton et al. to propose planktonic shedding as a key survival mechanism of bacterial biofilms (84). Common pathogens have been found in nasopharyngeal biofilms and MEEs of children with RAOM (86) and chronic OME (Sheyn et al., unpublished). These findings are consistent with planktonic shedding from the nasopharynx into the middle ear is a likely mechanism for RAOM and OME pathogenesis and may explain the high frequency of negative MEF cultures and frequent recurrence of these diseases.

### Detection and treatment of biofilms

Commonly used modalities to detect and measure biofilms include CSLM, SEM, fluorescent *in situ* hybridization (FISH), lectin-binding, and immunohistochemical techniques (75–78, 80, 82, 83, 87). These methods require an adequate tissue sample, thus restricting the study of middle ear biofilms to animal models and the study of adenoid biofilms to patients undergoing adenoidectomy, while *in vivo* studies are all but impossible.

Optical coherence tomography (OCT) is an emerging imaging modality which may allow non-invasive, *in vivo* detection of middle ear biofilms. This technology uses near-infrared laser waves to penetrate tissue to produce live, three-dimensional images (88), in a manner similar to ultrasound. However, the shorter wavelength of near-infrared waves compared to ultrasound waves permits sub-micrometer resolution. Like ultrasound, OCT is ideal for use in children because it is well-tolerated, causes no tissue injury, and avoids radiation exposure. Notably, the depth of penetration is limited to 1–2 mm due to scattering artifact. OCT has been used to detect retinal disease in multiple sclerosis (89) and age-related macular degeneration (90), yet its clinical applications may extend to a variety of medical fields (91). Nguyen et al recently demonstrated OCT-based *in vivo* detection of middle ear biofilms in adults with chronic OM (92). In a follow-up study, OCT findings were directly correlated with acoustic measurements of the TM in a similar adult population (93). These early studies were limited in sample size and further investigation is ongoing. OCT remains a highly promising non-invasive method of detecting biofilms which may play a role in diagnosing biofilm-related diseases in the middle ear.

Tympanostomy tubes likely alter middle ear flora by providing ventilation and increased oxygen tension. The impact on biofilm behavior is unclear, but may affect detachment rates (94). As a foreign body, tympanostomy tubes promote biofilm growth in the setting of initial infection by acting as a scaffold for bacterial colonization and extracellular matrix formation. Tube material may be an important factor in biofilm development. An *in vivo* animal study found that among several materials, only ion-bombarded silicone tubes prevented *S. aureus* biofilm adherence (95), although this effect was not seen with *P. aeruginosa* (96). Recently, the protective effect of coated silicone tubes has been investigated in several *in vitro* studies. MRSA is inhibited by vancomycin-coated tubes (97). *P. aeruginosa* biofilms are inhibited by polyvinylpyrrolidone (PVP)-coated tubes (98) and piperacillin-tazobactam coated tubes (99). Silver oxide-coated tubes, which reduce postoperative otorrhea (100), do not seem to resist biofilms (97, 99). Further research may lead to culture-directed selection of coated tympanostomy tubes to eradicate OM. The risk of selecting for drug resistant strains through low dose antimicrobial therapy remains a concern.

Middle ear biofilms are not eradicated by commonly prescribed topical antimicrobials (101). Agents that are effective in treating upper respiratory tract biofilms, such as mupirocin and gentian violet (102), are difficult to deliver or potentially ototoxic within the middle ear. Proposed strategies focus on electromechanical and biochemical disruption of biofilm adherence and proliferation.

Pulsed laser therapy has been demonstrated to dislodge middle ear biofilms by generating shockwaves (103). Electromagnetic, ultrasonic, and photo-therapy may enhance antimicrobial delivery or activity in certain applications (104).

Biochemical disruption includes identification of specific molecular targets (87), enhanced drug delivery (104, 105), and disruption of quorum sensing (105). Drug carriers such as liposomes (106) and biocompatible polymers (104) may offer a way to bypass the protective extracellular matrix, allowing penetration and controlled delivery of antimicrobials directly into the biofilm.

Quorum sensing is a complex process which allows cell-to-cell communication between biofilm bacteria. Extracellular DNA, released by cell autolysis, is a method of genetic exchange and is required for biofilm formation (107). DNase has been studied as a way to destroy this free-floating DNA and thus inhibit quorum sensing (108). Alginate lyase targets a key element of the *P. aeruginosa* extracellular matrix and disrupts existing biofilms (109). Naturally occurring compounds such as bacterial proteins (110) and tea-tree oil (111) may also possess antibiofilm properties.

Bakaletz described early research on several promising vaccines which have shown good efficacy in preventing OM as well as *in vitro* studies demonstrating eradication of existing biofilms (87). The possibility of preventative and therapeutic vaccines to target middle ear biofilms may dramatically change the landscape of AOM.

## Conclusion – Implication of Biofilm Infection in the Pathogenesis of OM

Many of these recent studies have demonstrated that RAOM and COME like many other chronic infections in humans such as chronic tonsillitis, cholesteatoma, chronic rhinosinusitis, cystic fibrosis, catheter infections, and infections in implants such as heart valves may be partially explained by the persistent nature of biofilm phenotypes. By combining the classical theories of the pathogenesis of OM with new insights of the nature of biofilms infections we may be able to develop a more comprehensive understanding of OM. Indeed biofilms help to explain many previously documented observations regarding OM, i.e., MEEs that are culture negative and yet have bacterial RNA identified by PCR, persistence of middle ear infections despite appropriate level of therapeutic antibiotics, and the efficacy of low dose antibiotics in the incidence of RAOM.

Our recent work has evaluated the presence of middle ear pathogens by utilizing Real Time PCR in the MEF and adenoids of children with both RAOM and COME. We found that all MEFs contained middle ear pathogens and that every pathogen identified in the MEF was also identified in the matched adenoid specimens (Sheyn et al., unpublished). Although the N-numbers are small, this work suggests that biofilms may play a role in COME.

**Figure 1:**
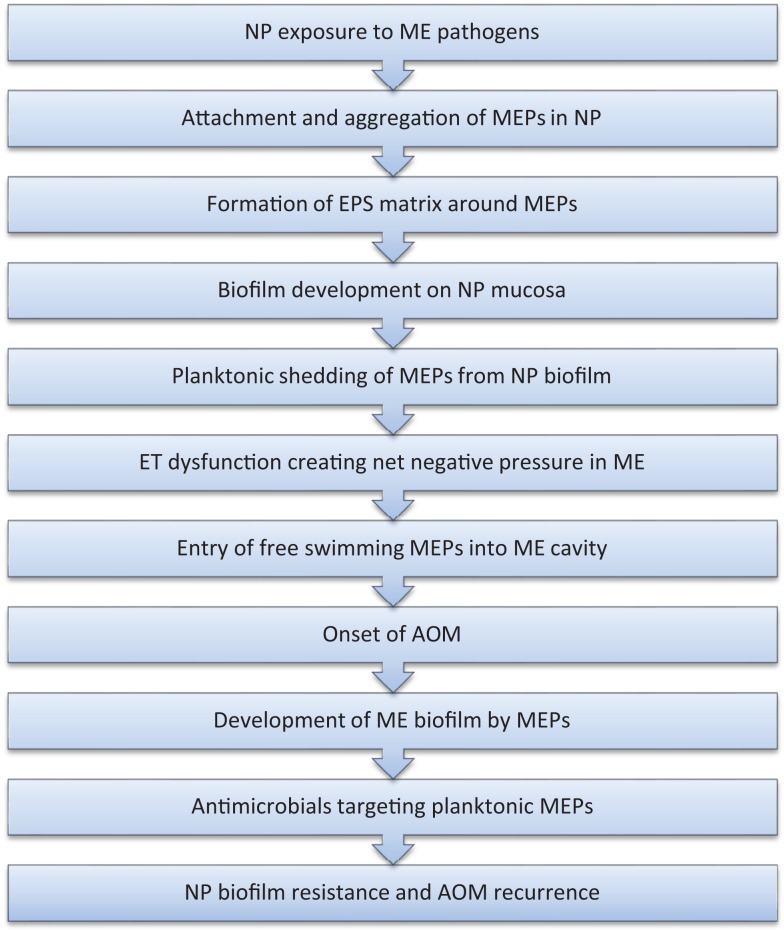
**Proposed algorithm for RAOM pathogenesis**.

The algorithm depicted in Figure 1 combines new concepts of biofilm infections with classical models of pathogenesis of OM. In this paradigm, the initial step in the development of RAOM is the exposure of the nasopharynx to known middle ear pathogens followed by colonization and subsequent biofilm formation by these bacteria. ET dysfunction in otitis prone children creates a net negative pressure in the middle ear and allows planktonically shed middle ear pathogens entry into the middle ear cavity. This is followed colonization and subsequent biofilm formation in the middle ear which results in the development of AOM. This episode of AOM is diagnosed by a primary care practitioner and is usually treated with a short course of antimicrobials. Although the patient may show clinical improvement, presumably due to effective antibiotic treatment of planktonic organisms in the middle ear, the nasopharyngeal biofilm remains resistant and it may again shed planktonic organisms to reinfect the middle ear cavity, resulting in and thus explaining the recurrence of AOM.

Research over the past several decades has elucidated anatomical factors important in the pathogenesis of OM in children. Although there has also been significant research on the identification of common pathogens including *H. influenzae*, *S. pneumoniae*, and *M. catarrhalis*, the mechanisms these pathogens utilize to colonize and persist in the host have not been well described. Biofilm phenotypes, as previously discussed, have been shown to resist host defenses through several mechanisms and persist despite therapeutic levels of antimicrobials. These characteristics of biofilm infection may help explain recurrent and persistent nature of many infectious entities. Therefore, a more comprehensive understanding of the host-organism via biofilm phenotypes in RAOM and COME expand our knowledge of the underlying pathogenesis of these recurrent and chronic disease states. This information could serve as a springboard in the development of new animal models of OM, new imaging techniques in diagnosis and novel therapeutic interventions for treatment.

## Conflict of Interest Statement

The authors declare that the research was conducted in the absence of any commercial or financial relationships that could be construed as a potential conflict of interest.

## References

[1] Rovers MM Schilder AG Zielhuis GA Rosenfeld RM Otitis media. Lancet (2004) 363(9407):465–73 10.1016/S0140-6736(04)15495-0 14962529

[2] Bluestone C Definitions, terminology, and classification. In: Rosenfeld RM Bluestone C, editors. Evidence-Based Otitis Media. Hamilton, ON: BC Decker (1999). p. 85–103

[3] American Academy of Pediatrics Subcommittee on Management of Acute Otitis Media Diagnosis and management of acute otitis media. Pediatrics (2004) 113(5):1451–65 10.1542/peds.113.5.1451 15121972

[4] Coker TR Chan LS Newberry SJ Limbos MA Suttorp MJ Shekelle PG Diagnosis, microbial epidemiology, and antibiotic treatment of acute otitis media in children: a systematic review. JAMA (2010) 304(19):2161–9 10.1001/jama.2010.1651 21081729

[5] McCaig LF Besser RE Hughes JM Trends in antimicrobial prescribing rates for children and adolescents. JAMA (2002) 287(23):3096–102 10.1001/jama.287.23.3096 12069672

[6] Arguedas A Kvaerner K Liese J Schilder AG Pelton SI Otitis media across nine countries: disease burden and management. Int J Pediatr Otorhinolaryngol (2010) 74(12):1419–24 10.1016/j.ijporl.2010.09.022 20965578

[7] Teele DW Klein JO Rosner B Epidemiology of otitis media during the first seven years of life in children in greater Boston: a prospective, cohort study. J Infect Dis (1989) 160(1):83–94 10.1093/infdis/160.1.83 2732519

[8] Hoberman A Marchant CD Kaplan SL Feldman S Treatment of acute otitis media consensus recommendations. Clin Pediatr (Phila) (2002) 41(6):373–90 10.1177/000992280204100602 12166789

[9] Bondy J Berman S Glazner J Lezotte D Direct expenditures related to otitis media diagnoses: extrapolations from a pediatric medicaid cohort. Pediatrics (2000) 105(6):E72 10.1542/peds.105.6.e72 10835085

[10] Gates GA Cost-effectiveness considerations in otitis media treatment. Otolaryngol Head Neck Surg (1996) 114(4):525–30 10.1016/S0194-5998(96)70243-7 8643261

[11] Daly KA Hoffman HJ Kvaerner KJ Kvestad E Casselbrant ML Homoe P Epidemiology, natural history, and risk factors: panel report from the ninth international research conference on otitis media. Int J Pediatr Otorhinolaryngol (2010) 74(3):231–40 10.1016/j.ijporl.2009.09.006 19836843

[12] Black S Shinefield H Fireman B Lewis E Ray P Hansen JR Efficacy, safety and immunogenicity of heptavalent pneumococcal conjugate vaccine in children. Northern California Kaiser Permanente Vaccine Study Center Group. Pediatr Infect Dis J (2000) 19(3):187–95 10.1097/00006454-200003000-00003 10749457

[13] Taylor S Marchisio P Vergison A Hausdorff WP Haggard M Pneumococcal conjugate vaccines and otitis media. Int J Otolaryngol (2012) 2012:764573 10.1371/journal.pone.0075558 23209472 PMC3502885

[14] Taylor S Marchisio P Vergison A Harriague J Hausdorff WP Haggard M Impact of pneumococcal conjugate vaccination on otitis media: a systematic review. Clin Infect Dis (2012) 54(12):1765–73 10.1093/cid/cis292 22423134 PMC3357481

[15] Hoppe HL Johnson CE Otitis media: focus on antimicrobial resistance and new treatment options. Am J Health Syst Pharm (1998) 55(18):1881–97 9784768 10.1093/ajhp/55.18.1881

[16] Coco AS Horst MA Gambler AS Trends in broad-spectrum antibiotic prescribing for children with acute otitis media in the United States, 1998-2004. BMC Pediatr (2009) 9:41 10.1186/1471-2431-9-41 19552819 PMC2711950

[17] Goossens H Ferech M Vander Stichele R Elseviers M ESAC Project Group. Outpatient antibiotic use in Europe and association with resistance: a cross-national database study. Lancet (2005) 365(9459):579–87 10.1016/S0140-6736(05)70799-6 15708101

[18] McCormick AW Whitney CG Farley MM Lynfield R Harrison LH Bennett NM Geographic diversity and temporal trends of antimicrobial resistance in *Streptococcus pneumoniae* in the United States. Nat Med (2003) 9(4):424–30 10.1038/nm839 12627227

[19] Tan TQ Antibiotic resistant infections due to *Streptococcus pneumoniae*: impact on therapeutic options and clinical outcome. Curr Opin Infect Dis (2003) 16(3):271–7 10.1097/00001432-200306000-00015 12821820

[20] Mera RM Miller LA Daniels JJ Weil JG White AR Increasing prevalence of multidrug-resistant *Streptococcus pneumoniae* in the United States over a 10-year period: Alexander Project. Diagn Microbiol Inf Dis (2005) 51(3):195–200 10.1016/j.diagmicrobio.2004.10.009 15766606

[21] Jacobs MR Good CE Beall B Bajaksouzian S Windau AR Whitney CG Changes in serotypes and antimicrobial susceptibility of invasive *Streptococcus pneumoniae* strains in Cleveland: a quarter century of experience. J Clin Microbiol (2008) 46(3):982–90 10.1128/JCM.02321-07 18234877 PMC2268364

[22] Joloba ML Windau A Bajaksouzian S Appelbaum PC Hausdorff WP Jacobs MR Pneumococcal conjugate vaccine serotypes of *Streptococcus pneumoniae* isolates and the antimicrobial susceptibility of such isolates in children with otitis media. Clin Infect Dis (2001) 33(9):1489–94 10.1086/323027 11588694

[23] CDC Office-related antibiotic prescribing for persons aged ≤14 years – United States, 1993–1994 to 2007–2008. MMWR Morb Mortal Wkly Rep (2011) 60(34):1153–6 21881545

[24] Farrell DJ Klugman KP Pichichero M Increased antimicrobial resistance among nonvaccine serotypes of *Streptococcus pneumoniae* in the pediatric population after the introduction of 7-valent pneumococcal vaccine in the United States. Pediatr Infect Dis J (2007) 26(2):123–8 10.1097/01.inf.0000253059.84602.c3 17259873

[25] Vergison A Microbiology of otitis media: a moving target. Vaccine (2008) 26(Suppl 7):G5–10 10.1016/j.vaccine.2008.11.006 19094935 PMC7127463

[26] Laine MK Tähtinen PA Ruuskanen O Huovinen P Ruohola A Symptoms or symptom-based scores cannot predict acute otitis media at otitis-prone age. Pediatrics (2010) 125(5):e1154–61 10.1542/peds.2009-2689 20368317

[27] Lieberthal AS Carroll AE Chonmaitree T Ganiats TG Hoberman A Jackson MA The diagnosis and management of acute otitis media. Pediatrics (2013) 131(3):e964–99 10.1542/peds.2012-3488 23439909

[28] Shaikh N Hoberman A Kaleida PH Rockette HE Kurs-Lasky M Hoover H Otoscopic signs of otitis media. Pediatr Infect Dis J (2011) 30(10):822–6 10.1097/INF.0b013e31822e6637 21844828

[29] Kvaerner KJ Tambs K Harris JR Magnus P Distribution and heritability of recurrent ear infections. Ann Otol Rhinol Laryngol (1997) 106(8):624–32 9270423 10.1177/000348949710600802

[30] Rye MS Warrington NM Scaman ES Vijaysekaran S Coates HL Anderson D Genome-wide association study to identify the genetic determinants of otitis media susceptibility in childhood. PLoS One (2012) 7(10):e48215 10.1371/journal.pone.0048215 23133572 PMC3485007

[31] Corbeel L What is new in otitis media? Eur J Pediatr (2007) 166(6):511–9 10.1007/s00431-007-0461-8 17364173 PMC1876255

[32] Bluestone CD Epidemiology and pathogenesis of chronic suppurative otitis media: implications for prevention and treatment. Int J Pediatr Otorhinolaryngol (1998) 42(3):207–23 10.1016/S0165-5876(97)00147-X 9466224

[33] Revai K Dobbs LA Nair S Patel JA Grady JJ Chonmaitree J Incidence of acute otitis media and sinusitis complicating upper respiratory tract infection: the effect of age. Pediatrics (2007) 119(6):e1408–12 10.1542/peds.2006-2881 17545367

[34] Malik V Verma RU Joshi V Sheehan PZ An evidence-based approach to the 12-min consultation for a child with Down’s syndrome. Clin Otolaryngol (2012) 37(4):291–6 10.1111/j.1749-4486.2012.02482.x 22925092

[35] Sheahan P Miller I Sheahan JN Earley MJ Blayney AW Incidence and outcome of middle ear disease in cleft lip and/or cleft palate. Int J Pediatr Otorhinolaryngol (2003) 67(7):785–93 10.1016/S0165-5876(03)00098-3 12791455

[36] Kitajiri M Sando I Hashida Y Doyle WJ Histopathology of otitis media in infants with cleft and high-arched palates. Ann Otol Rhinol Laryngol (1985) 94(1 Pt 1):44–50 4038594 10.1177/000348948509400110

[37] Aniansson G Svensson H Becker M Ingvarsson L Otitis media and feeding with breast milk of children with cleft palate. Scand J Plast Reconstr Surg Hand Surg (2002) 36(1):9–15 10.1080/028443102753478318 11925834

[38] Sando I Takahashi H Otitis media in association with various congenital diseases. Preliminary study. Ann Otol Rhinol Laryngol Suppl (1990) 148:13–6 2140931 10.1177/00034894900990s605

[39] Skoner AR Skoner KR Skoner DP Allergic rhinitis, histamine and otitis media. Allergy Asthma Proc (2009) 30(5):470–81 10.2500/aap.2009.30.3272 19843400

[40] Heikkinen T Chonmaitree T Importance of respiratory viruses in acute otitis media. Clin Microbiol Rev (2003) 16(2):230–41 10.1128/CMR.16.2.230-241.2003 12692096 PMC153141

[41] Kleemola M Nokso-Koivisto J Herva E Syrjänen R Lahdenkari M Kilpi T Is there any specific association between respiratory viruses and bacteria in acute otitis media of young children? J Infect (2006) 52(3):181–7 10.1016/j.jinf.2005.05.012 15992930 PMC7173109

[42] Brook I Effects of exposure to smoking on the microbial flora of children and their parents. Int J Pediatr Otorhinolaryngol (2010) 74(5):447–50 10.1016/j.ijporl.2010.01.006 20129680

[43] Paradise JL Rockette HE Colborn DK Bernard BS Smith CG Kurs-Lasky M Otitis media in 2253 Pittsburgh-area infants: prevalence and risk factors during the first two years of life. Pediatrics (1997) 99(3):318–33 10.1542/peds.99.3.318 9041282

[44] Daly KA Brown JE Lindgren BR Meland MH Le CT Giebink GS Epidemiology of otitis media onset by six months of age. Pediatrics (1999) 103(6 Pt 1):1158–66 10.1542/peds.103.6.1158 10353923

[45] Froom J Culpepper L Green LA de Melker RA Grob P Heeren T A cross-national study of acute otitis media: risk factors, severity, and treatment at initial visit. Report from the International Primary Care Network (IPCN) and the Ambulatory Sentinel Practice Network (ASPN). J Am Board Fam Pract (2001) 14(6):406–17 11757882

[46] Ladomenou F Kafatos A Tselentis Y Galanakis E Predisposing factors for acute otitis media in infancy. J Infect (2010) 61(1):49–53 10.1016/j.jinf.2010.03.034 20394772

[47] Rovers MM Zielhuis GA Ingels K van der Wilt GJ Day-care and otitis media in young children: a critical overview. Eur J Pediatr (1999) 159(1):1–6 9950299 10.1007/pl00021272

[48] Macintyre EA Karr CJ Koehoorn M Demers P Tamburic L Lencar C Otitis media incidence and risk factors in a population-based birth cohort. Paediatr Child Health (2010) 15(7):437–42 21886448 10.1093/pch/15.7.437 PMC2948776

[49] Bluestone CD Klein JO Otitis media with effusion, atelectasis, and Eustachian tube dysfunction. In: Bluestone CD Stool SE, editors. Pediatric Otolaryngology. Philadelphia: Saunders (1983). p. 356–512

[50] Bylander-Groth A Stenström C Eustachian tube function and otitis media in children. Ear Nose Throat J (1998) 77(9):762–4 9787519

[51] Bluestone CD Klein JO Otitis Media in Infants and Children. Philadelphia: Saunders (1988).

[52] Ingelstedt S Ivarsson A Jonson B Quantitative determination of tubal ventilation during changes in ambient pressure as during ascent and descent in aviation. Acta Otolaryngol (1967) 228(Suppl):31

[53] Hergils L Magnuson B Regulation of negative middle ear pressure without tubal opening. Arch Otolaryngol Head Neck Surg (1988) 114(12):1442–4 10.1001/archotol.1988.01860240092030 3190873

[54] Politzer A Diseases of the Ear. 4th ed Philadelphia: Lea Brothers & Co (1903).

[55] Shupak A Attias J Aviva J Melamed Y Oxygen diving-induced middle ear under-aeration. Acta Otolaryngol (1995) 115(3):422–6 10.3109/00016489509139341 7653265

[56] Beuerlein M Nelson RN Welling DB Inner and middle ear hyperbaric oxygen-induced barotrauma. Laryngoscope (1997) 107(10):1350–6 10.1097/00005537-199710000-00011 9331312

[57] Miura MS Mascaro M Rosenfeld RM Association between otitis media and gastroesophageal reflux: a systematic review. Otolaryngol Head Neck Surg (2012) 146(3):345–52 10.1177/0194599811430809 22157391

[58] Al-Saab F Manoukian JJ Al-Sabah B Almot S Nguyen LH Tewfik TL Linking laryngopharyngeal reflux to otitis media with effusion: pepsinogen study of adenoid tissue and middle ear fluid. J Otolaryngol Head Neck Surg (2008) 37(4):565–71 19128594

[59] McCoul ED Goldstein NA Koliskor B Weedon J Jackson A Goldsmith AJ A prospective study of the effect of gastroesophageal reflux disease treatment on children with otitis media. Arch Otolaryngol Head Neck Surg (2011) 137(1):35–41 10.1001/archoto.2010.222 21242544

[60] Giebink GS Otitis media: the chinchilla model. Microb Drug Resist (1999) 5(1):57–72 10.1089/mdr.1999.5.57 10332723

[61] Watanabe N DeMaria TF Lewis DM Mogi G Lim DJ Experimental otitis media in chinchillas. II. Comparison of the middle ear immune responses to *S pneumonia* types 3 and 23. Ann Otol Rhinol Laryngol Suppl (1982) 93:9–16 6807184

[62] Giebink GS The pathogenesis of pneumococcal otitis media in chinchillas and the efficacy of vaccination in prophylaxis. Rev Infect Dis (1981) 3(2):342–53 7256090 10.1093/clinids/3.2.342

[63] Sadé J The nasopharynx, Eustachian tube and otitis media. J Laryngol Otol (1994) 108(2):95–100 7802761 10.1017/s0022215100126003

[64] Brook I Shah K Jackson W Microbiology of healthy and diseased adenoids. Laryngoscope (2000) 110(6):994–9 10.1097/00005537-200006000-00021 10852520

[65] Yang YP Loosmore SM Underdown BJ Klein MH Nasopharyngeal colonization with nontypeable *Haemophilus influenzae* in chinchillas. Infect Immun (1998) 66(5):1973–80 9573078 10.1128/iai.66.5.1973-1980.1998 PMC108152

[66] Syrjanen RK The value of nasopharyngeal culture in predicting the etiology of acute otitis media in children less than two years of age. Pediatr Infect Dis J (2006) 25(11):1032–6 10.1097/01.inf.0000241097.37428.1d 17072126

[67] Brook I Gober AE In vitro bacterial interference in the nasopharynx of otitis media-prone and non-otitis media prone children. Arch Otolaryngol Head Neck Surg (2000) 126(8):1011–3 10.1001/archotol.126.8.1011 10922236

[68] Hall-Stoodley L Stoodley P Evolving concepts in biofilm infections. Cell Microbiol (2009) 11(7):1034–43 10.1111/j.1462-5822.2009.01323.x 19374653

[69] Fux CA Costerton JW Stewart PS Stoodley P Survival strategies of infectious biofilms. Trends Microbiol (2005) 13(1):34–40 10.1016/j.tim.2004.11.010 15639630

[70] Stewart PS Costerton JW Antibiotic resistance of bacteria in biofilms. Lancet (2001) 358(9276):135–8 10.1016/S0140-6736(01)05321-1 11463434

[71] Mah TF O’Toole GA Mechanisms of biofilm resistance to antimicrobial agents. Trends Microbiol (2001) 9(1):34–9 10.1016/S0966-842X(00)01913-2 11166241

[72] Potera C Forging a link between biofilms and disease. Science (1999) 283(5409):1837 10.1126/science.283.5409.1837 10206887

[73] Zuliani G Carron M Gurrola J Coleman C Haupert M Berk R Identification of adenoid biofilms in chronic rhinosinusitis. Int J Pediatr Otorhinolaryngol (2006) 70(9):1613–7 10.1016/j.ijporl.2006.05.002 16781783

[74] Bakaletz LO Bacterial biofilms in otitis media: evidence and relevance. Pediatr Infect Dis J (2007) 26(10 Suppl):S17–9 10.1097/INF.0b013e318154b273 18049376

[75] Post JC Direct evidence of bacterial biofilms in otitis media. Laryngoscope (2001) 111(12):2083–94 10.1097/00005537-200112000-00001 11802002

[76] Ehrlich GD Veeh R Wang X Costerton JW Hayes JD Hu FZ Mucosal biofilm formation on middle-ear mucosa in the chinchilla model of otitis media. JAMA (2002) 287(13):1710–5 10.1001/jama.287.13.1710 11926896

[77] Hall-Stoodley L Hu FZ Gieseke A Nistico L Nguyen D Hayes J Direct detection of bacterial biofilms on the middle-ear mucosa of children with chronic otitis media. JAMA (2006) 296(2):202–11 10.1001/jama.296.2.202 16835426 PMC1885379

[78] Thornton PB Rigby P Wiertsema SP Filion P Langlands J Coates HL Multi-species bacterial biofilm and intracellular infection in otitis media. BMC Pediatr (2011) 11:94 10.1186/1471-2431-11-94 22018357 PMC3224757

[79] Barakate M Beckenham E Curotta J da Cruz M Bacterial biofilm adherence to middle-ear ventilation tubes: scanning electron micrograph images and literature review. J Laryngol Otol (2007) 121(10):993–7 17553184 10.1017/S0022215107008870

[80] Hoa M Syamal M Sachdeva L Berk R Coticchia J Demonstration of nasopharyngeal and middle ear mucosal biofilms in an animal model of acute otitis media. Ann Otol Rhinol Laryngol (2009) 118(4):292–8 19462851 10.1177/000348940911800410

[81] Zuliani G Carlisle M Duberstein A Haupert M Syamal M Berk R Biofilm density in the pediatric nasopharynx: recurrent acute otitis media versus obstructive sleep apnea. Ann Otol Rhinol Laryngol (2009) 118(7):519–24 19708492 10.1177/000348940911800711

[82] Hoa M Syamal M Schaeffer MA Sachdeva L Berk R Coticchia J Biofilms and chronic otitis media: an initial exploration into the role of biofilms in the pathogenesis of chronic otitis media. Am J Otolaryngol (2010) 31(4):241–5 10.1016/j.amjoto.2009.02.015 20015753

[83] Nistico L Kreft R Gieseke A Coticchia JM Burrows A Khampang P Adenoid reservoir for pathogenic biofilm bacteria. J Clin Microbiol (2011) 49(4):1411–20 10.1128/JCM.00756-10 21307211 PMC3122793

[84] Costerton JW Nickel JC Ladd TI Suitable methods for the comparative study of free-living and surface-associated bacterial populations. In: Poindexter JS Leadbetter ER, editors. Methods and Special Applications in Bacterial Ecology. New York: Plenum (1986). 3 p.

[85] Costerton JW Lewandowski Z Caldwell DE Korber DR Lappin-Scott HM Microbial films. Annu Rev Microbiol (1995) 49:711–45 10.1146/annurev.mi.49.100195.003431 8561477

[86] Duberstein A Hoa M Christensen L Berk RS Coticchia JM Comparison of middle ear pathogens in nasopharyngeal biofilms and middle ear effusions of children with recurrent acute otitis media. Poster Session Presented at Combined Otolaryngological Spring Meetings. Orlando, FL: (2008).

[87] Bakaletz LO Bacterial biofilms in the upper airway – evidence for role in pathology and implications for treatment of otitis media. Paediatr Respir Rev (2012) 13(3):154–9 10.1016/j.prrv.2012.03.001 22726871 PMC3509202

[88] Huang D Swanson EA Lin CP Schuman JS Stinson WG Chang W Optical coherence tomography. Science (1991) 254(5035):1178–81 10.1126/science.1957169 1957169 PMC4638169

[89] Dörr J Wernecke KD Bock M Gaede G Wuerfel JT Pfueller CF Association of retinal and macular damage with brain atrophy in multiple sclerosis. PLoS One (2011) 6(4):e18132 10.1371/journal.pone.0018132 21494659 PMC3072966

[90] Keane PA Patel PJ Liakopolous S Heussen FM Sadda SR Tufail A Evaluation of age-related macular degeneration with optical coherence tomography. Surv Ophthalmol (2012) 57(5):389–414 10.1016/j.survophthal.2012.01.006 22898648

[91] Zysk AM Nguyen FT Oldenburg AL Marks DL Boppart SA Optical coherence tomography: a review of clinical development from bench to bedside. J Biomed Opt (2007) 12(5):051403 10.1117/1.2793736 17994864

[92] Nguyen CT Jung W Kim J Chaney EJ Novak M Stewart CN Noninvasive in vivo optical detection of biofilm in the human middle ear. Proc Natl Acad Sci U S A (2012) 109(24):9529–34 10.1073/pnas.1201592109 22645342 PMC3386096

[93] Nguyen CT Robinson SR Jung W Novak MA Boppart SA Allen JB Investigation of bacterial biofilm in the human middle ear using optical coherence tomography and acoustic measurements. Hear Res (2013) 301:193–200 10.1016/j.heares.2013.04.001 23588039 PMC3669223

[94] Thormann KM Saville RM Shukla S Spormann AM Induction of rapid detachment in *Shewanella oneidensis* MR-1 biofilms. J Bacteriol (2005) 187(3):1014–21 10.1128/JB.187.3.1014-1021.2005 15659679 PMC545703

[95] Saidi IS Biedlingmaier JF Whelan P In vivo resistance to bacterial biofilm formation on tympanostomy tubes as a function of tube material. Otolaryngol Head Neck Surg (1999) 120(5):621–7 10.1053/hn.1999.v120.a94162 10229584

[96] Jang CH Cho YB Choi CH Effect of ion-bombarded silicone tympanostomy tube on ciprofloxacin-resistant *Pseudomonas aeruginosa* biofilm formation. Int J Pediatr Otorhinolaryngol (2012) 76(10):1471–3 10.1016/j.ijporl.2012.06.025 22819832

[97] Jang CH Park H Cho YB Choi CH Effect of vancomycin-coated tympanostomy tubes on methicillin-resistant *Staphylococcus aureus* biofilm formation: in vitro study. J Laryngol Otol (2010) 124(6):594–8 10.1017/S0022215109992672 20056010

[98] Ojano-Dirain CP Silva RC Antonelli PJ Biofilm formation on coated silicone tympanostomy tubes. Int J Pediatr Otorhinolaryngol (2013) 77(2):223–7 10.1016/j.ijporl.2012.10.027 23200869

[99] Jang CH Park H Cho YB Choi CH Park IY The use of piperacillin-tazobactam coated tympanostomy tubes against ciprofloxacin-resistant *Pseudomonas* biofilm formation: an in vitro study. Int J Pediatr Otorhinolaryngol (2009) 73(2):295–9 10.1016/j.ijporl.2008.10.020 19095310

[100] Chole RA Hubbell RN Antimicrobial activity of silastic tympanostomy tubes impregnated with silver oxide. Arch Otolaryngol Head Neck Surg (1995) 121(5):562–5 10.1001/archotol.1995.01890050054010 7727091

[101] Oxley KS Thomas JG Ramadan HH Effect of ototopical medications on tympanostomy tube biofilms. Laryngoscope (2007) 117(10):1819–24 10.1097/MLG.0b013e3180d09ede 17690613

[102] Smith A Buchinsky FJ Post JC Eradicating chronic ear, nose, and throat infections: a systematically conducted literature review of advances in biofilm treatment. Otolaryngol Head Neck Surg (2011) 144(3):338–47 10.1177/0194599810391620 21493193

[103] Krespi YP Stoodley P Hall-Stoodley L Laser disruption of biofilm. Laryngoscope (2008) 118(7):1168–73 10.1097/MLG.0b013e31816ed59d 18401277

[104] Smith AW Biofilms and antibiotic therapy: is there a role for combating bacterial resistance by the use of novel drug delivery systems? Adv Drug Deliv Rev (2005) 57(10):1539–50 10.1016/j.addr.2005.04.007 15950314

[105] Vlastarakos PV Nikolopoulos TP Maragoudakis P Tzagaroulakis A Ferekidis E Biofilms in ear, nose, and throat infections: how important are they? Laryngoscope (2007) 117(4):668–73 10.1097/MLG.0b013e318030e422 17415138

[106] Jones MN Song YH Kaszuba M Reboiras MD The interaction of phospholipid liposomes with bacteria and their use in the delivery of bactericides. J Drug Target (1997) 5(1):25–34 10.3109/10611869708995855 9524311

[107] Whitchurch CB Tolker-Nielsen T Ragas PC Mattick JS Extracellular DNA required for bacterial biofilm formation. Science (2002) 295(5559):1487 10.1126/science.295.5559.1487 11859186

[108] Hall-Stoodley L Nistico L Sambanthamoorthy K Dice B Nguyen D Mershon WJ Characterization of biofilm matrix, degradation by DNase treatment and evidence of capsule downregulation in *Streptococcus pneumoniae* clinical isolates. BMC Microbiol (2008) 8:173 10.1186/1471-2180-8-173 18842140 PMC2600794

[109] Lamppa JW Griswold KE Alginate lyase exhibits catalysis-independent biofilm dispersion and antibiotic synergy. Antimicrob Agents Chemother (2013) 57(1):137–45 10.1128/AAC.01789-12 23070175 PMC3535929

[110] Dusane DH Damare SR Nancharaiah YV Ramaiah N Venugopalan VP Kumar AR Disruption of microbial biofilms by an extracellular protein isolated from epibiotic tropical marine strain of *Bacillus licheniformis*. PLoS One (2013) 8(5):e64501 10.1371/journal.pone.0064501 23691235 PMC3655075

[111] Park H Jang CH Cho YB Choi CH Antibacterial effect of tea-tree oil on methicillin-resistant *Staphylococcus aureus* biofilm formation of the tympanostomy tube: an in vitro study. In vivo (2007) 21(6):1027–30 18210750

